# Computational discovery and systematic analysis of protein entangling motifs in nature: from algorithm to database†

**DOI:** 10.1039/d4sc08649j

**Published:** 2025-03-31

**Authors:** Puqing Deng, Yuxuan Zhang, Lianjie Xu, Jinyu Lyu, Linyan Li, Fei Sun, Wen-Bin Zhang, Hanyu Gao

**Affiliations:** a Department of Chemical and Biological Engineering, Hong Kong University of Science and Technology Clear Water Bay Hong Kong hanyugao@ust.hk; b Beijing National Laboratory for Molecular Sciences, Key Laboratory of Polymer Chemistry & Physics of Ministry of Education, Center for Soft Matter Science and Engineering, College of Chemistry and Molecular Engineering, Peking University Beijing 100871 P. R. China wenbin@pku.edu.cn; c Department of Data Science, City University of Hong Kong Kowloon Hong Kong; d AI for Science (AI4S)-Preferred Program, Shenzhen Graduate School, Peking University Shenzhen 518055 P. R. China

## Abstract

Nontrivial protein topology has the potential to revolutionize protein engineering by enabling the manipulation of proteins' stability and dynamics. However, the rarity of topological proteins in nature poses a challenge for their design, synthesis and application, primarily due to the limited number of available entangling motifs as synthetic templates. Discovering these motifs is particularly difficult, as entanglement is a subtle structural feature that is not readily discernible from protein sequences. In this study, we developed a streamlined workflow enabling efficient and accurate identification of structurally reliable and applicable entangling motifs from protein sequences. Through this workflow, we automatically curated a database of 1115 entangling protein motifs from over 100 thousand sequences in the UniProt Knowledgebase. In our database, 73.3% of C2 entangling motifs and 80.1% of C3 entangling motifs exhibited low structural similarity to known protein structures. The entangled structures in the database were categorized into different groups and their functional and biological significance were analyzed. The results were summarized in an online database accessible through a user-friendly web platform, providing researchers with an expanded toolbox of entangling motifs. This resource is poised to significantly advance the field of protein topology engineering and inspire new research directions in protein design and application.

## Abstract 2

We developed an integrated computational algorithm for the rapid discovery of protein entangling motifs from sequences. This algorithm identified diverse and novel motifs, which have been summarized and made accessible through a web server.

## Graphical abstract

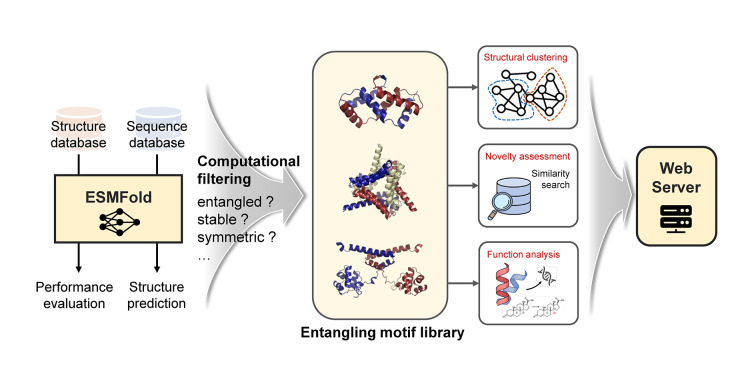

## Introduction

Understanding protein topology is crucial for advancing protein engineering and synthetic biology. Topology, a mathematical discipline describing the spatial properties of objects preserved under continuous deformations like twisting and stretching but without tearing, has been applied to the chemical field. This foundational concept was first introduced into the chemical field by Frisch and Wasserman in 1961 (ref. 1) to describe the spatial invariant of molecules and later stretched to a broader scope concerning connectivity and spatial relationships among molecular segments.^2^ For decades, a wide range of highly complex topologies in small molecules have been successfully achieved, including the Star of David,^3^ prime double trefoil link,^4^ 819 knot,^5^*etc.* In biomacromolecules like DNA, intricate topologies involving chain underwinding, overwinding, tangling and knotting are common.^6^

In contrast, only a limited number of natural proteins exhibit nontrivial topologies.^7^ Nonlinear proteins with such unconventional topologies are called ‘topological proteins’.^8^ These proteins exhibit unique properties, including enhanced stability, controllable quaternary structures, dynamic switching properties, and a synergistic multivalency effect.^7^ The ability to artificially synthesize topological proteins holds promise for achieving functionalities that linear proteins cannot provide.

The “assembly-reaction” synergy was proposed as an effective approach for integrating unconventional topologies into proteins. This method involves a preorganization of nascent proteins into genetically encoded intertwined structures – referred to as entangling motifs – with well-defined spatial orientations followed by a covalent ligation to mechanically lock the topological structures. Through this method, some relatively simple topologies have been derived including protein [2]catenanes,^9–11^ protein heterocatenanes,^12–14^ higher-order [*n*]catenanes,^15,16^ star proteins^17^ and lasso proteins.^18^ The currently used entangling motifs for this approach are still limited to relatively simple types including p53dim, HP0242 and artificial entangled structures obtained by rethreading, which also limits the complexity and diversity of achievable protein topologies.^19–21^ Expanding the database of entangling motifs can enrich the toolbox of entangled assemblies and advance the design and synthesis of topological proteins.

Existing databases, such as KnotProt,^22^ LinkProt^23^ and AlphaKnot,^24^ have been developed to catalogue entangled proteins. However, these databases were not specially curated for the purpose of topological protein synthesis, and instances of interchain entanglement within symmetric assemblies are rare. Our previous work has curated a library of known symmetric entangling motifs from the Protein Data Bank (PDB),^20^ a database of proteins with known structures. In this work, different symmetric types have been taken into consideration, as highly ordered, large-scale oligomeric assemblies such as protein cages can be formed by arranging subunits with simple symmetries, including C2, C3, and C4, into a well-defined dihedral angle.^25^ Here, our focus shifts to the far larger sequence database with unknown assembly structures. The UniProt Knowledgebase (UniProtKB)^26^ now consists of around 250 million entries and is 1000 times larger than the PDB and contains much more sequence and functional information to investigate. In addition to symmetric oligomers, heterodimers are also valuable for expanding the designable topology space. As an example, a cross-entwining heterodimer motif derived from p53dim paved the way for the synthesis of higher-order protein catenanes.^15^ By sequence screening, we envision an extensive entangling motif database that includes both symmetric oligomers and heterodimers, with more diverse entangled structures as well as reflecting a deeper understanding of their associated biological significance.

In this work, we developed a streamlined workflow empowered by deep learning to quickly and accurately identify promising entangling motifs from protein sequences. Upon receiving the input sequences, our workflow performed necessary duplications or linkages based on the selected oligomeric state, preparing them for multimeric structure prediction. We utilized ESMFold,^27^ a deep learning model enabling a large-scale structure prediction, to efficiently predict the tertiary structures of the assemblies. These predicted structures underwent a series of automated curation steps to detect entanglements as well as ensuring their reliability and applicability in topological protein synthesis. Sequence screening over 100 thousand sequences from the UniProtKB through our developed workflow culminated in the establishment of a database of 1115 entangling motifs, including C2 homodimers, C3 homotrimers and heterodimers. Our systematic analysis of this database provided valuable insights into the structural commonalities and functional significance of entangled structures. Additionally, we have created a web platform to provide easy access to this valuable toolbox, thereby facilitating advancements in protein topology engineering and deepening our understanding of the biological roles of entangled structures.

## Results and discussion

### Evaluating the performance of ESMFold in discovering entangling motifs

Although ESMFold has been extensively evaluated for its atomic accuracy in predicting protein tertiary structures,^27^ its ability to detect entangled structures has not yet been explored. While there may be some correlation between these two tasks, predicting tertiary structures focuses on the deviation of positions for each atom, while identifying entangled structures requires accurate prediction of the relative positions of different chains. Therefore, it is essential to investigate how well ESMFold can discover intended entangling motifs of different types before sequence screening. The evaluation findings would not only offer us a general idea of the reliability of our predicted database but also guide our following screening scope and filtration criteria.

To evaluate ESMFold, we leveraged experimentally resolved protein structures deposited in the PDB. We retrieved four sub-datasets, namely PDB-Heterodim, PDB-C2, PDB-C3, and PDB-C4/D2, which consist of heterodimers, homodimers with C2 symmetry, homotrimers with C3 symmetry, and homotetramers with C4 or D2 symmetry, respectively. Multimer structures were predicted by adding a soft linker composed of 25 consecutive glycine residues between subunits, and their extent of chain entanglement was quantitatively assessed using the Gauss Linking Number (GLN). The GLN was originally introduced as a numerical invariant that describes the linking of two closed curves.^28^ When applied to two open protein backbones, it is no longer topologically invariant but still measures the degree of intertwining, with a larger |GLN| indicating greater entanglement.^20,29^ Compared to other entanglement detection methods that require random chain closures,^23,30^ the GLN for open chains is less computationally demanding and more suitable for rapid systematic mining. Although highly entangled chains can occasionally have a GLN of 0 (*e.g.*, Whitehead link), such cases are rare in protein assemblies, as suggested by LinkProt statistics.^23^ For assemblies with more than two chains, we calculated |GLN| among all possible combinations of subunit pairs and selected the highest as the representative. In this study, we measured the accuracy of ESMFold in predicting the |GLN| of protein assemblies with regression metrics like absolute errors and squared errors. We also measured how well ESMFold could distinguish between entangled and unentangled structures with classification metrics by setting a |GLN| threshold of 0.7. Assemblies with a |GLN| greater than or equal to 0.7 were classified as entangled, while those below the threshold were considered unentangled. The ground truth GLN in the evaluation datasets and ESMFold prediction results are shown in Fig. S1 and S2.†

**Fig. 1:**
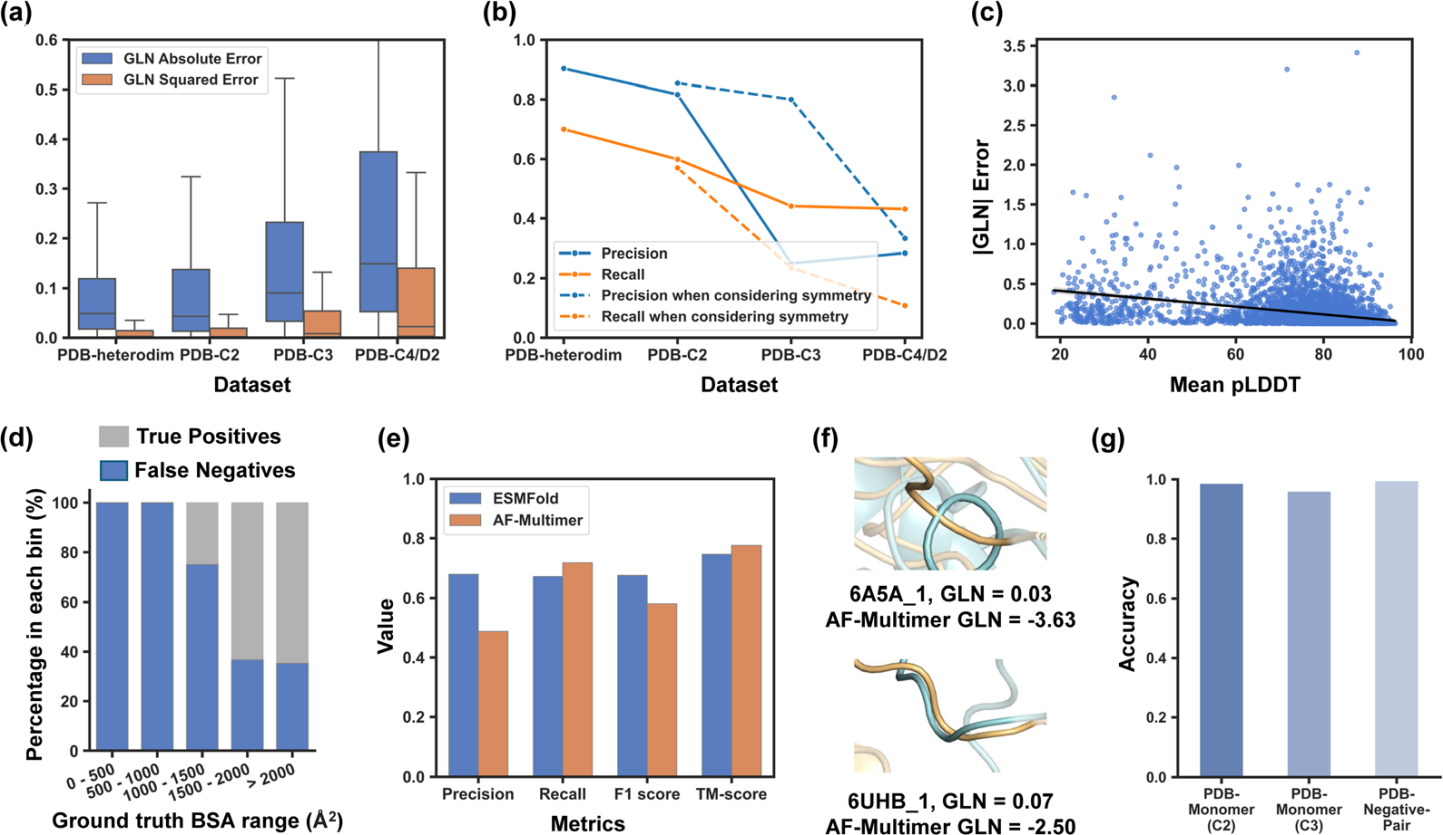
Evaluation of ESMFold in capturing entangled protein assemblies. (a) GLN absolute error and squared error for different datasets; (b) binary classification metrics for different datasets by setting a |GLN| threshold of 0.7. The dotted curve gives the precision and recall when considering a predicted structure with both |GLN| ≥ 0.7 and the corresponding symmetry as positive; (c) correlation between |GLN| error and mean pLDDT on the PDB-C2 dataset with a negative linear fit; (d) percentage of true positives and false negatives for different ground truth buried surface area (BSA) ranges; (e) comparison of ESMFold and AF-multimer on the PDB-Recent dataset; (f) examples of AF-Multimer predictions where different subunits overlap. (g) The accuracy of ESMFold in classifying non-interacting protein chains as negatives (|GLN| of predicted assembly structures < 0.7 or 0.5). PDB-Monomer (C2) refers to repeating the monomer sequence twice to predict homodimer structures. PDB-Monomer (C3) refers to repeating the monomer sequence three times to predict homotrimer structures. PDB-Negative-Pair refers to predicting the assembly structures of non-interacting sequence pairs.

The results showed that ESMFold's capability to identify entanglement in complexes decreased from PDB-Heterodim to PDB-C2 to PDB-C3 to PDB-C4/D2 datasets, with an increasing GLN absolute error and a decreasing precision and recall (as shown in Fig. 1a and b). This trend was consistent with previous studies on structure prediction, where accuracy tended to decrease as the number of chains modelled increased.^31–33^ As the primary goal of this study was to create a reliable database of entangling motifs, the focus was on precision rather than recall. ESMFold demonstrated high precision in capturing entangled structures in heterodimers and homodimers (0.903 and 0.816, respectively). For PDB-C3, the precision reached up to 0.8 when only the predicted structures with a GLN greater than or equal to 0.7 and C3 symmetry were considered positive. However, precision for PDB-C4/D2 was low. Based on these findings, ESMFold was used to screen the sequence database to identify entangling motifs of heterodimers, homodimers, and homotrimers in this study. Despite high screening precision, it is worth noting that some genuinely entangled structures may be predicted with low GLN values and consequently excluded during the screening process. Our tests revealed that 30.0% of truly entangled heterodimers were overlooked, along with 43.0% of homodimers and 76.5% of homotrimers when considering symmetries. Further identification of these mistakenly excluded entangling motifs remains an outstanding challenge.

Upon closer examination of the evaluation results on the PDB-C2 dataset, a moderate negative correlation was observed between |GLN| error and the mean predicted local distance difference test (pLDDT), a confidence metric produced by ESMFold (Fig. 1c). Predicted structures with a higher mean pLDDT tended to have a lower |GLN| error. However, the correlation was not quite strong, and some large GLN errors still existed even in structures with a high pLDDT. Additionally, an increased true positive rate was observed with an increase in the buried surface area (BSA), indicating that entangled structures with a higher BSA were more likely to be correctly identified (Fig. 1d). The reason for this could be that entanglement in structures with a smaller BSA was less stable and more susceptible to the influence of the linker that was added during the prediction process. These findings motivated us to establish filtration criteria for both pLDDT and BSA in our subsequent sequence screening to further enhance the reliability of our final database.

The performance of ESMFold was compared to that of AF-Multimer^34^ with an auxiliary dataset named PDB-Recent that was composed of homodimers released after 2018/4/30, getting rid of multimers in the training set of AF-Multimer. Since ESMFold was trained solely on monomer structures,^27^ there should also be no information leakage regarding how two chains interact or whether they entangle between ESMFold's training set and the PDB-Recent dataset. Though it predicted with a higher template modelling score^35^ (TM-score, a measurement of similarity between predicted and ground truth protein structures), AF-Multimer demonstrated lower precision and F1 score for identifying entangled structures. AF-Multimer had a higher tendency to produce overlapping residues between different subunits, which would lead to a high |GLN| and produce false positives. Some overlapping examples are shown in Fig. 1f.

The previously discussed datasets evaluated ESMFold's ability to identify entangled structures when prior knowledge of protein chain interactions was available. However, this information is not accessible during the actual sequence screening process, where we arbitrarily assume that sequences will form protein assemblies, which is often not the case. Therefore, we constructed a PDB-Monomer dataset consisting of 1568 monomers without assembly structures in the PDB to assess if ESMFold could effectively handle monomers when predicting homomeric assemblies (including homodimers and homotrimers), classifying them as negative examples (|GLN| of predicted structures < 0.7 or 0.5). Additionally, we randomly sampled 2000 protein pairs, termed PDB-Negative-Pair, from a dataset derived by Cong *et al.*,^36^ which included protein pairs from two different complexes, with no experimental evidence of interactions between them. We used ESMFold to predict heterodimer structures formed by these sequence pairs and evaluated its ability to classify these non-interactions as negatives (|GLN| of predicted structures < 0.7). As a result, ESMFold demonstrated strong performance in identifying negative homodimers, negative homotrimers, and negative sequence pairs, achieving accuracies of 0.987, 0.960, and 0.995, respectively (Fig. 1g). These findings support the feasibility of our screening algorithm, even in the absence of prior knowledge regarding interactions between different chains.

## Screening for entangling motifs

To prepare protein sequences for the screening, a collection of 105 567 sequences from all strains (*e.g.* K-12, O157:H7, and NU14) of *Escherichia coli* (*E. coli*) was retrieved from UniProtKB after redundancy removal. *E. coli* is a bacterium commonly used for recombinant protein synthesis because of its fast growth rate, ease of genetic manipulation, and cost-effectiveness, which shall facilitate experimental validation and use of the discovered entangling motifs. The length distribution of retrieved sequences is shown in Fig. S3.†

**Fig. 2:**
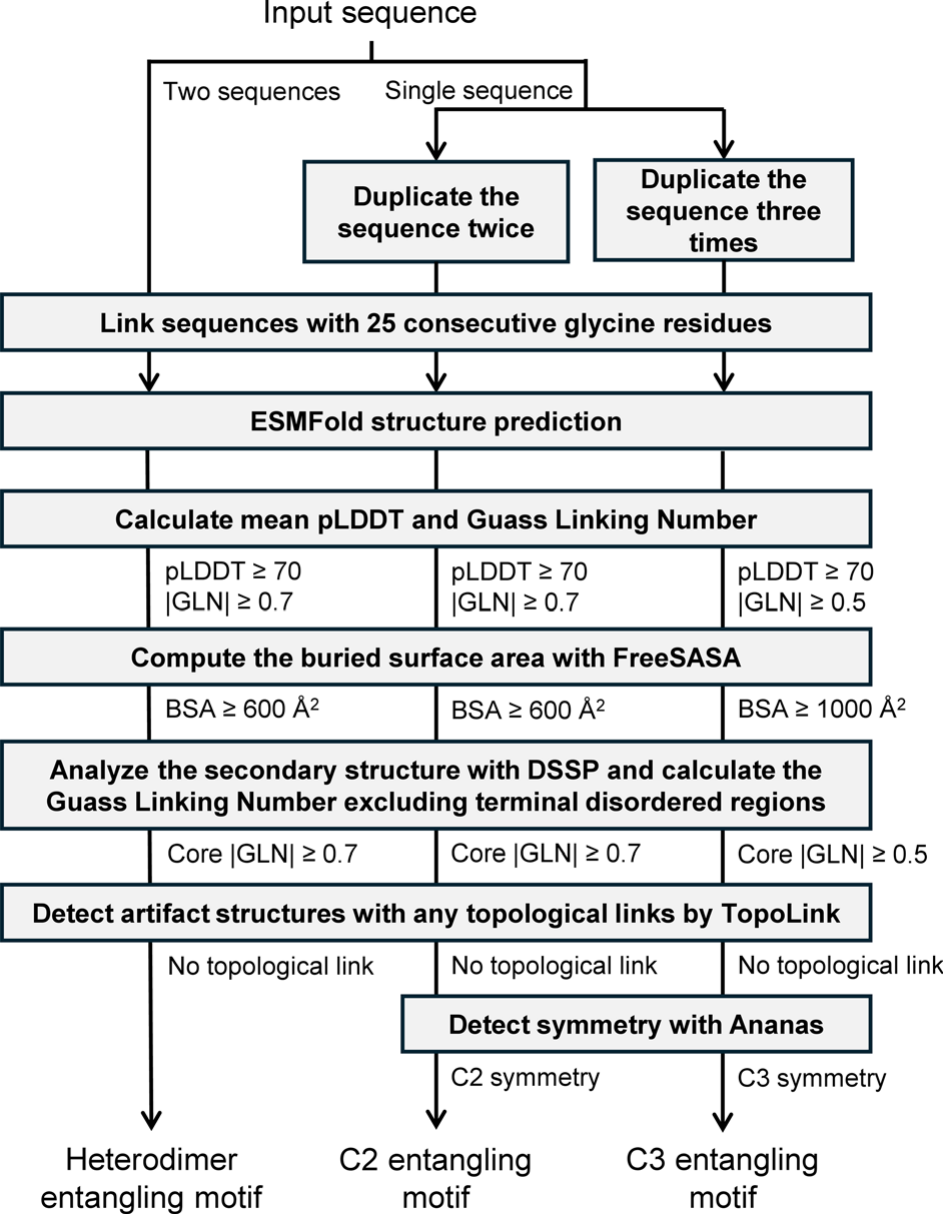
Streamlined workflow for identifying promising protein entangling motifs with reliability and applicability.

Our sequence screening workflow is illustrated in Fig. 2. To screen for C2 and C3 motifs, each sequence was duplicated two or three times, respectively, under the assumption that they could self-assemble into dimers or trimers. The resulting sequences were then linked with 25 glycine residues before being fed into the ESMFold model. For the screening of heterodimers, the vast search space of over 10 billion sequence pairs resulting from the combination of every two sequences in the collection of 105 567 sequences was deemed intractable. To address this issue, we limited the selection of sequences to those with a length between 50 and 250 and randomly paired them with no repeats. This pairing process was repeated five times, resulting in a total of 227 130 sequence pairs. Although this still represents a very small fraction of the combinatorial space, we anticipated discovering some new heterodimer entangling motifs from this searching space within the computational limits of our resources.

In our sequence screening, we did not explicitly verify that the screened sequences or sequence pairs would form protein assemblies. However, based on our previous test results with PDB-Monomer and PDB-Negative-Pair, ESMFold demonstrated a strong ability to implicitly identify non-interacting assemblies and classify them as negative.

The pLDDT and GLN distributions of predicted structures are shown in Fig. S4 and S5,† respectively. After obtaining the predicted structures, we sequentially applied a filtering process based on the following criteria:

(1) |GLN| ≥ 0.7 or 0.5. We applied a |GLN| cut-off of 0.7 to dimers to distinguish between entangled and non-entangled structures, and 0.5 to trimers considering their smaller population and the fact that for certain topologies, such as Borromean rings,^37^ a high degree of intertwining is not indispensable, although a certain level of intertwining is still required.^20^

(2) pLDDT ≥ 70 for more reliable structure predictions.

(3) For dimers, we requested a BSA ≥ 600 Å^2^. For trimers, we set a stricter threshold of 1000 Å^2^. A larger BSA ensured sufficient stability for topology synthesis as well as a higher prediction accuracy as previously discussed.

(4) Core |GLN| ≥ 0.7. Core GLN refers to the GLN calculated when excluding the terminal disordered regions in all subunits in this study. Disordered regions are flexible regions without regular secondary structures, where backbone chains are often highly dynamic and can adopt multiple conformations.^38^ Entanglements formed by these regions are not reliable enough for topology synthesis.

(5) Desired symmetries such as C2 and C3 were requested.

(6) A previous study indicated that AF-Multimer produced false topological links whose formation required the unfolding of protein chains, which was nearly impossible in the experimental structures.^39^ The same problem was observed for ESMFold as well in our experiments, necessitating the detection and disposal of structures with such topological links.

Some unqualified structure examples discarded during the filtration are shown in Fig. S6.† The number of entries left during each curation process is shown in Table S1.† Ultimately, 962 C2 homodimers, 141 C3 homotrimers, and 12 heterodimers were identified.

### Identifying entangling motifs with novel structures or sequences

**Fig. 3:**
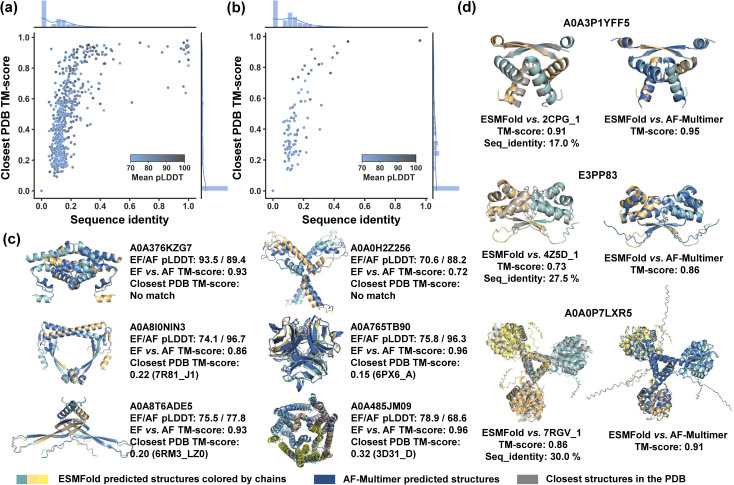
Structure and sequence similarity analysis between discovered entangling motifs and known structures in PDB. (a) Closest PDB TM-scores of all C2 entangling motifs *versus* the corresponding sequence identity. Both TM-scores and sequence identities are given corresponding to the aligned segment. (b) Closest PDB TM-scores of all C3 entangling motifs *versus* the corresponding sequence identity. Both TM-scores and sequence identities are given corresponding to the aligned segment. (c) Examples of motifs with low structure and sequence similarity with known structures in the PDB. EF and AF refer to ESMFold and AF-Multimer, respectively. Closest PDB TM-score was searched based on ESMFold-predicted structures. (d) Examples of motifs with low sequence identity but high structure similarity with known structures in the PDB. The TM-score in (c) and (d) was given by MM-align^42^ and the sequence identity corresponded to the aligned segment. These predicted structures were further verified by AF-Multimer (dark blue).

Our sequence screening is expected to yield a more diversified range of entwined assembly structures that go beyond our current knowledge of protein structures, thereby enhancing the designability of protein topologies. To validate this and facilitate the data organization experimental utilization of these structures as well, we evaluate the structural novelty of discovered entangling motifs (Fig. 3). Specifically, we aimed to identify novel structures in our discovered entangling motifs by comparing their structures with previously known structures in the PDB using Foldseek,^40^ a fast protein structure alignment tool. Plots of the closest PDB TM-score *versus* corresponding sequence identity for C2 and C3 entangling motifs (Fig. 3a and b) share a similar pattern with little chance that two close sequences fold into distinct structures, but some cases where different sequences correspond to similar structures. With a minimum alignment coverage of 0.7, Foldseek reported 73.3% (705 motifs) of C2 motifs without a similar known structure in the PDB (closest PDB TM-score ≥ 0.5), among which 59.7% (421 motifs) found no structural match (closest PDB TM-score = 0). For C3 entangling motifs, Foldseek reported 80.1% (113 motifs) without a similar known structure in the PDB, with 43.4% (49 motifs) having no structural match. This suggested that a great fraction of discovered entangled structures were new compared to the known ones. Some examples of these motifs are shown in Fig. 3c and their structures were verified by AF-Multimer. Additionally, many discovered motifs with highly similar structures in the PDB were reported with no similar sequences (Fig. 3a, b and d). For example, the predicted dimer structure of the UniProtKB sequence A0A3P1YFF5 highly resembled 2CPG_1 (TM-score 0.91) in the PDB but a BLASTp^41^ search returned no hits for similar sequences in the PDB. Likewise, the UniProtKB sequence E3PP83 had no hits reported by the BLASTp sequence search, yet it was predicted to have a similar structure to 4Z5D_1 (TM-score 0.73). These results demonstrated that our entangling motif database extended the current knowledge on both the sequence and structure of entangled structures, thereby broadening the available building tools for topology synthesis.

### Clustering by local entangled structure similarity

**Fig. 4:**
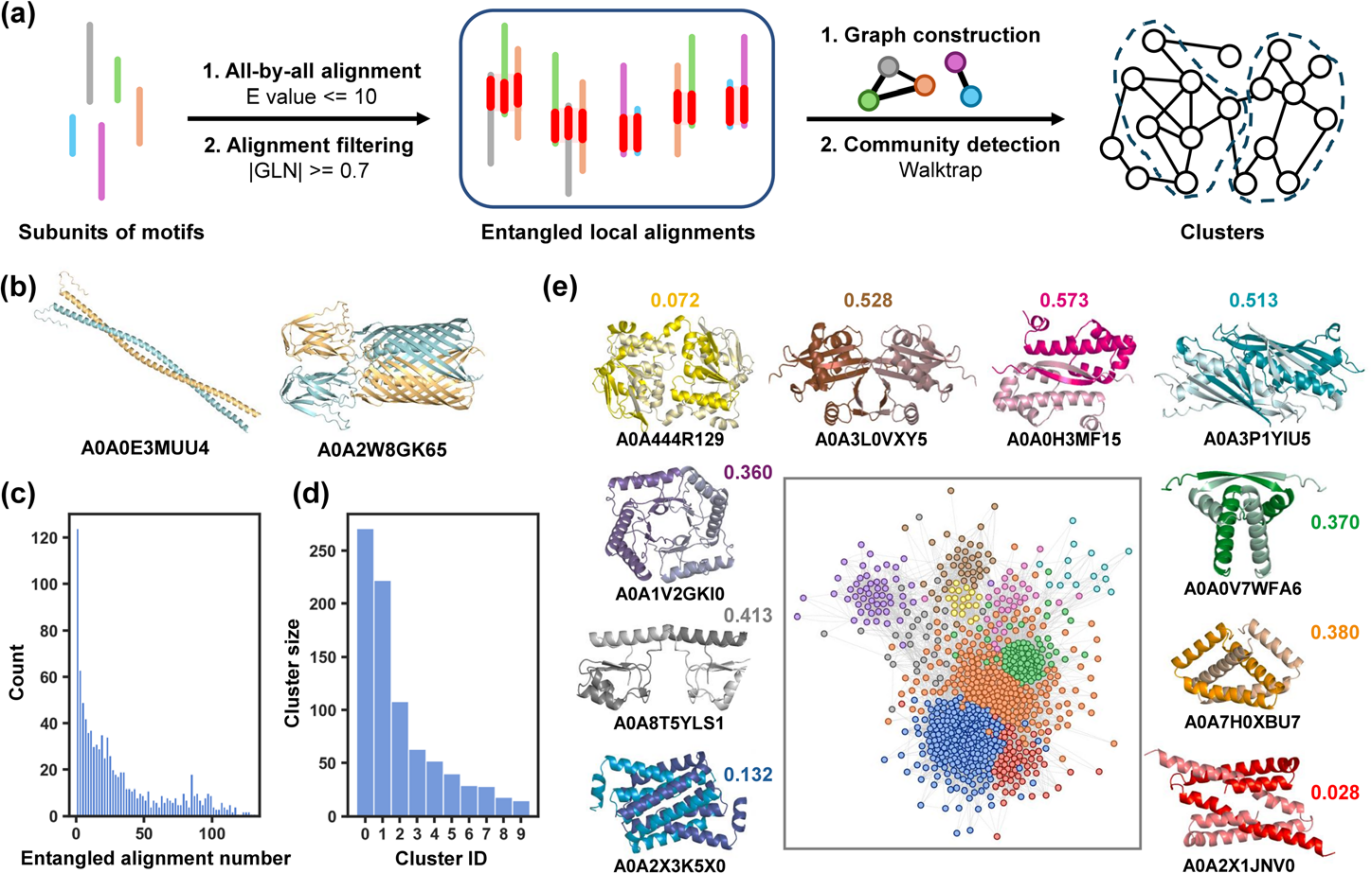
C2 motif clustering by local entangled structure similarity. (a) Schematic of clustering steps. (b) Examples of motifs with few (0 or 1) entangled alignments. (c) Distribution of entangled alignment numbers for each motif. (d) Sizes of clusters with a size larger than 15. (e) Visualization of the clustering graph network in which closer nodes represent higher structure similarity. Different clusters are marked in different colours. A representative motif is given for each cluster. Coloured numbers are averaged closest PDB TM-scores of entangling motifs in each cluster, demonstrating the relative novelty of each motif type.

Structural clustering could endow us with a deeper understanding of the structural motifs in which entanglements frequently occur. The Foldseek clustering algorithm grouped structures by global similarity. However, entanglements can take place locally in only a portion of a protein, such as within a single domain. Motifs with different global structures but similar entangled cores should be clustered into one group (as illustrated in Fig. S7†). To address this, we clustered the discovered entangling motifs based solely on their local entangled sites by adapting a previous approach described for domain prediction^43^ (Fig. 4a). We extended this method from monomers to symmetric oligomers (C2 and C3 motifs) by extracting one subunit for clustering. We also incorporated an alignment filtration step after the all-by-all structural similarity search, in which alignments corresponding to non-entangled domains were excluded. Following filtration, no motifs were observed possessing more than one entangled domain, which made it reasonable to let each node represent one motif in the graph network.

Out of 962 C2 entangling motifs, 124 (12.9%) motifs ended up with no or only one alignment (Fig. 4c), which accounts for the resulting singleton clusters. Through a manual examination, these motifs were mainly of two categories. The first consists coiled-coils in which two or more alpha-helical peptides are wrapped around each other in a parallel or antiparallel manner to form a supercoil^44,45^ (Fig. 4b, left). These structural motifs were found less likely to be aligned with each other owing to different twisting extents characterized by parameters like pitch lengths and associated pitch angles.^45^ The other consists domain-swapping dimers, where two identical monomers exchange their “domains” or structural units connected by a hinge loop to form a dimer containing two monomer units that are structurally similar to the original monomer^46,47^ (Fig. 4b, right). These entanglements were achieved by structural units across different domains and thus not captured by Foldseek, which aligned structures within one domain, and they have already been well investigated in many previous studies.^48–52^

The clustering of C2 entangling motifs yielded 102 clusters, with 10 clusters having a size greater than or equal to 15, covering 89.40% (860 motifs) of all C2 motifs (Fig. 4d). Representative structures of the top ten largest clusters are visualized in Fig. 4e, revealing a wide range of structural diversity. The most prevalent entangling motif was the helix up-down bundle,^53^ which consisted of several alpha helices packed together nearly parallelly or antiparallelly, accounting for 34.7% of all C2 motifs (Fig. 4e, blue and red). This large population suggested that the intermolecular interactions between parallel or antiparallel helices could easily link two helix bundles into an intertwined pattern. Indeed, some researchers *de novo* designed protein dimers through domain swapping between four-helix bundles for higher stability.^54,55^ In the second largest group, entanglements were formed by the interaction of two two-helix or three-helix orthogonal motifs (Fig. 4e, orange). Ribbon-helix-helix^56^ was another typical intertwined motif, taking up a proportion of 11.2% (Fig. 4e, green). Additionally, structure units such as the alpha/beta barrel^57^ (Fig. 4e, purple), two intertwined beta strands (Fig. 4e, pink), hotdog folds^58,59^ in which sausage-like long central helices are wrapped around by highly distorted six-stranded beta sheets (Fig. 4e, cyan), and others also participated in the C2 entangling family. It is worth noting that our structural discussions here focused solely on the entangled sites of the assemblies. There were numerous types of structural motifs connected to the entangled sites in our database that were not the focus of our discussion (Fig. S7†).

**Fig. 5:**
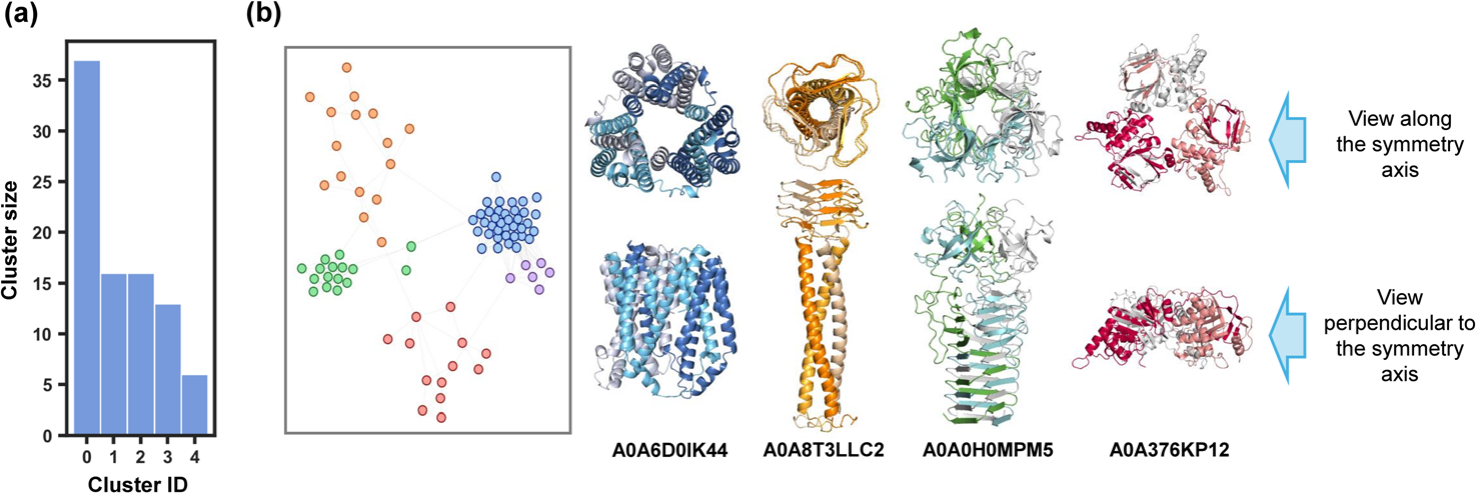
C3 motif clustering by local entangled structure similarity. (a) Sizes of clusters with a size larger than 5. (b) Visualization of the clustering graph network in which closer nodes represent higher structure similarity. Different clusters are marked in different colours. Some representative motifs for different clusters are given.

The same clustering steps were applied to organize the discovered C3 entangling motifs. Compared with C2 entangling motifs, in which case 87.94% of structures were clustered into large groups (group size ≥ 15), C3 entangling motifs demonstrate more diverse entangling patterns, with 5 out of 52 clusters containing more than 5 motifs, covering only 62.41% of the whole (Fig. 5a). Helix up-down bundles retain a dominant population, accounting for 26.2% (37 motifs) of all C3 motifs. Besides, three chain intertwining could also be formed within a coiled coil motif (orange), beta orthogonal prism motif (green) and alpha-beta sandwiches (red) (Fig. 5b).

### Biological significance analysis

Natural proteins evolved over time to form essential structures that engage in various biological processes. A database of these entangled assemblies provides opportunities to understand the functional origin of protein chain intertwining. We mapped all entangled sequences to their functions by retrieving their Gene Ontology (GO) annotations^60,61^ related to molecular functions from the UniProtKB (Fig. S8 and S9†).

**Fig. 6:**
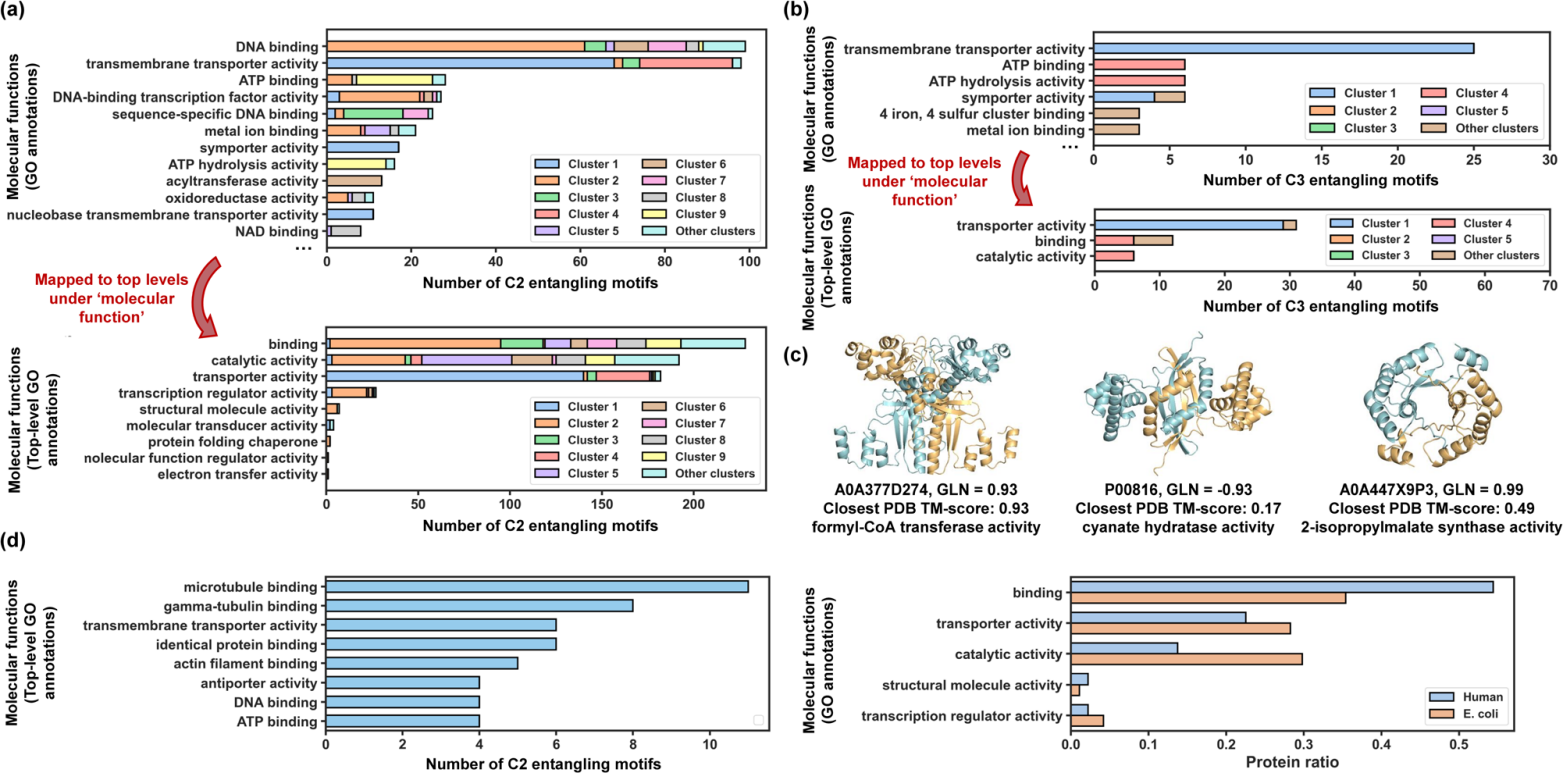
Biological significance of entangling motifs. (a) Top: top 12 most popular GO annotations for discovered C2 entangling motifs in terms of their populations. Sequences from different clusters are coloured by different colours, the same colours as those in Fig. 4e. Bottom: top-level terms mapped from all GO annotations in terms of their populations. (b) Top: top 6 most popular GO annotations for discovered C3 entangling motifs in terms of their populations. Sequences from different clusters are coloured by different colours, the same colours as those in Fig. 5b. Bottom: top-level terms mapped from all GO annotations in terms of their populations. (c) Examples of discovered entangling motifs with catalytic activity. (d) Molecular functions of entangling motifs from humans.

Out of 962 C2 entangling motifs, 525 were successfully mapped into 686 GO annotations covering 159 annotation types in total, which shows a wide range of possible functionalities for entangled structures. The annotation population demonstrated a highly skewed distribution, with the top 12 most popular annotation types (Fig. 6a, top) accounting for 61.5% of all annotations and the other 38.5% distributing over 147 annotation types. Among these annotations, DNA binding and transmembrane transporter activity have far larger populations than other annotation types, accounting for 17.8% and 15.0% of all annotations, respectively. These retrieved annotations are not mutually exclusive but follow certain hierarchy relationships structured as an acyclic directed graph with ‘child’ nodes being more specialized than their ‘parent’ nodes.^61^ To take a broad view of functional distributions, we mapped retrieved GO annotations to the top-level terms just below the root term ‘molecular_function’ based on the hierarchy graph (Fig. 6a, bottom). The mapping identified 9 broad functional categories for C2 entangling motifs, with 4 categories having much larger populations: binding (35.4%), catalytic activity (29.8%), transporter activity (28.2%) and DNA-binding transcription activity (4.2%). As for C3 entangling motifs, 60 out of 141 have GO annotations in the UniProtKB database, with the majority (51.7%) belonging to the transporter activity category (Fig. 6b). These results indicate that chain entanglements in both C2 and C3 assemblies are strongly correlated with specific functions, particularly binding, catalytic activity, and transporter activity.

Generally, chain intertwining in assembly structures enhances structural stability. For instance, entangling motifs featuring helix up-down bundles (coloured red and blue) are predominantly associated with transmembrane transporter activity for both C2 and C3 motifs. This aligns with previous findings that helix topologies are found prevalent in transmembrane transporter proteins^62^ and many transporters like potassium channel^63^ and Gltph^64^ are formed with multiple identical subunits assembled into symmetric homo-oligomers. The entanglements in these structures may further stabilize the transport channels. Besides, ribbon-helix-helix motifs (coloured green) mostly function as the DNA-binding protein, while motifs from cluster 9 (coloured yellow) typically exhibit both ATP binding and hydrolysis activity. Intertwining may serve to stabilize the binding interface^20,56^ while retaining certain flexibility, making binding proteins a large source of entanglements.

Chain entanglement may also act as a rigid spacer for regulating the orientations and positions of more than one functional domain. For example, entangling motifs in cluster 8 (coloured grey) are associated with NAD binding proteins. Each chain in these proteins binds to nicotinamide adenine dinucleotide with helix-turn-helix motifs and entanglements formed between two chains can serve as rigid spacers and dynamically regulate the spatial orientations of their binding sites (Fig. S10†).

Topological proteins could potentially contribute to enhanced stability compared with their linear counterparts,^7,65^ probably due to their more compact structures and limited side-chain conformations. Our study discovered a significant number of entangled homomeric enzymes with catalytic activity (29.8% of C2 motifs and 12.2% of C3 motifs) that could be potentially made into more stabilized catenanes through ‘assembly-reaction synergy’ without the need for extra entangled reaction motifs. To show some examples (Fig. 6c), the UniProtKB sequence P00816 annotated with cyanate hydratase activity (EC number: 4.2.1.104) was predicted adopt a highly intertwined homodimeric structure (GLN = −0.93). Cynase catalysed the degradation of cyanate to produce ammonia, a considerable alternative nitrogen source,^66^ and was regarded as a possible solution for reducing the environmental impact of cyanide.^67^ The sequence A0A447X9P3 with its homo-dimeric structure predicted to be entangled (GLN = 0.99) demonstrated 2-isopropylmalate synthase activity, which has practical applications in the food industry.^68^ These two example structures were novel enough to go beyond the realized protein structure scope, reported with a closest PDB TM-score of 0.17 and 0.49, respectively.

Using the same workflow as before, we identified 642 entangling motifs from approximately 50 000 human sequences to facilitate a functional comparison of these motifs across different proteomes (Fig. 6d). Notably, the two proteomes analysed exhibited the same top five broad functional categories, further confirming the correlation between these functions and inter-chain entanglement.

### Web server

We developed a web server, TangleDB (https://protein-database-sigma.vercel.app/), to compile information on the entangling motifs discussed in this study. TangleDB allows users to easily browse all available structures and search for specific ones based on various attributes, including GLN, pLDDT, symmetries, and proteomes. Additionally, TangleDB features a visualization interface, enabling users to quickly view multiple structures or conduct a detailed examination of individual structures.

## Methods

### Dataset

Six sub-datasets, namely PDB-Heterodim, PDB-C2, PDB-C3, PDB-C4/D2, PDB-Recent and PDB-Monomer, were downloaded from PDB with a subunit length ranging from 50 to 400 and a resolution smaller than 3.5 Å. We ensured that sequences in each dataset had a sequence similarity smaller than 0.4 by MMSeqs2 (ref. 69) clustering. The resulting dataset sizes are 1295, 5302, 891, 1149, 2375 and 1568 for PDB-Heterodim, PDB-C2, PDB-C3, PDB-C4/D2, PDB-Recent, and PDB-Monomer, respectively. PDB-Recent consisted of homodimers released between 2018/4/30 and 2023/08/23. An extra PDB-Negative-Pair dataset consisted of 2000 protein pairs with lengths ranging from 50 to 400, between which no experimental evidence of interactions was disclosed. These protein pairs were randomly sampled from a dataset derived by Cong *et al.*^36^

### GLN calculation

Generally, two protein chains with a more severe entanglement will result in a larger absolute GLN value. The GLN for two open backbones can be derived using
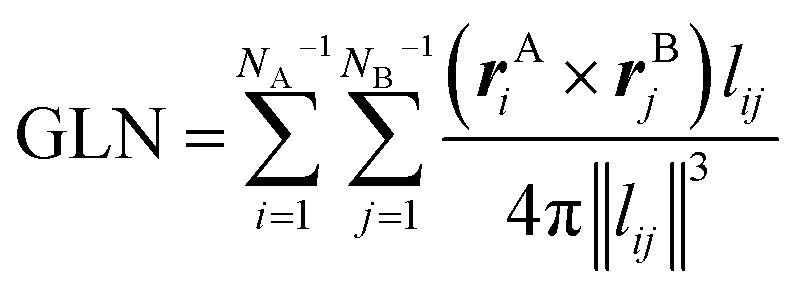
where ***r***^A^_*i*_ is the vector between the *i*^th^ and the (*i* + 1)^th^ α-carbon in chain A, pointing from the N-terminus to the C-terminus and ***r***^B^_*j*_ is the vector between the *j*^th^ and the (*j* + 1)^th^ α-carbon in chain B. GLN_*ij*_ is the GLN between any two vectors ***r***^A^_*i*_ and ***r***^B^_*j*_ from different protein chains. *N*_A_ and *N*_B_ denote the number of residues in chains A and B, respectively. ***l***_*ij*_ is the vector between the middle points of ***r***^A^_*i*_ and ***r***^B^_*j*_.

When encountering missing residues in downloaded PDB files, the coordinate records were indicated by blank lines and the GLN calculation ignored the gap regions by assuming that the missing residues contributed little to the mutual entanglements between the two chains and to avoid artificial introduction of possible entanglements.

### Sequence preparation for screening

Sequences in *E. coli* and humans with a length of 50 to 400 and released before 2023/07 were retrieved from the UniProtKB. To foster the sequence diversity, redundancy was removed using MMseqs2 (ref. 69) with a sequence identity cut-off of 40%. Sequences with their homodimers or homotrimers already deposited in the PDB were also discarded. This resulted in a final collection of 105 567 sequences in *E. coli* and 48 590 sequences in humans ready for screening.

### Structural analysis

The BSA was obtained by subtracting the average accessible surface area (ASA) of all subunits within the assembly from the ASA of the whole assembly. The ASA was calculated using FreeSASA.^70^ DSSP^71,72^ was used to analyse the secondary structures in predicted tertiary structures. Random coils were marked as blank in the DSSP files. Ananas^73^ was used to detect any symmetries from predicted tertiary structures with the maximum RMSD error set as 3 Å.

### Foldseek structure searching against the PDB

Foldseek decoded protein structures into the 3Di alphabet-like amino sequences, which consequently enabled fast structure similarity detection just like the way MMseqs2 did for sequence alignments. Though Foldseek was designed for monomer structure comparison, we applied it to a structure similarity search against the PDB for all our discovered C2 entangling motifs by only taking one subunit of the assembly as the query structure. This made sense as the assembly had C2 symmetry (the same for clustering). We searched for all similar structures in the PDB with a minimum alignment coverage of 0.7.

### Local entangled structure clustering

One subunit from each motif was extracted. The all-by-all alignment was applied by ‘foldseek easy-search’ with an *E*-value of 10. For all alignments, we calculated the corresponding |GLN| in the original homodimer and discarded those with a |GLN| smaller than the entanglement threshold (0.7 for dimers and 0.5 for trimers). When linking two motifs, we assigned a weight of 2 to the edge if alignments were captured no matter which motif was set as the query structure. Otherwise, we set the edge weight as one. The graph network clustering was applied using Walktrap.^74^ The graph network was visualized using graph-tool with the sfdp layout.^75^

## Conclusions

We have conducted a systematic search for diverse protein entangling motifs across the vast genomic space with our developed computational workflow. By inserting a soft linker between different monomers to predict assembly structures and applying GLN to evaluate the extent of chain entwining, ESMFold has shown high performance in discovering entangling motifs of heterodimers, C2 homodimers and C3 homotrimers from primary sequences. The precision of discovery can be further improved by applying filtration metrics such as symmetry, BSA and pLDDT. Through our curation pipeline, an entangling motif database with 962 C2 homodimers, 141 C3 homotrimers, and 12 heterodimers has consequently been established. Among the abundant homomeric entangling motifs, we have identified at least 818 (73.4%) novel structures with a TM-score smaller than 0.5 compared with any experimentally resolved structures. These entangled structural motifs were organized into clusters based on local entangled structure similarity, demonstrating a diverse range of entanglement patterns, with some occurring much more frequently than others. By analysing the correlation between entangled structures and GO annotations, we also reveal the biological significance of discovered entangling motifs. We believe that this expanded toolbox not only facilitates the design of protein topologies with higher complexity but also establishes a foundation for the development of innovative therapeutics and biomaterials utilizing topological proteins.

Notably, our work focused on the proteome from *E. coli* for better solubility, and there are likely manifold motifs in other proteomes that have yet to be identified. Additionally, due to the significantly large search space that exceeded our computational capacity, we employed a random pairing approach to reduce the search scope to a quite small fraction, resulting in a limited coverage. To address this limitation, faster computational methods^76,77^ that can identify potential protein–protein interactions could be utilized before applying our ESMFold-based filtration workflow. Our workflow could also be applied to existing protein interactome data^36,78^ for more efficient screening. Moreover, ESMFold exhibits poor performance in identifying entangling motifs with more subunits or higher-order symmetries, such as C4 and D2. Therefore, there is a need for the development of high-throughput algorithms to discover highly complex entangling motifs, which would further enhance the designable complexity of protein topologies.

## Data availability

Protein structure data for ESMFold performance evaluation, evaluation results, screening sequence data and all final motif structures are deposited in Zenodo (https://zenodo.org/records/14159943). The codes of our screening framework are publicly available at Zenodo (https://zenodo.org/records/14159943) and FigShare (https://doi.org/10.6084/m9.figshare.27922461.v1). Our web server can be accessed through https://protein-database-sigma.vercel.app/ and the related source codes are available at FigShare (https://doi.org/10.6084/m9.figshare.28019861.v1) and GitHub (https://github.com/AlanZhang-2468/protein-database.git).

## Author contributions

Puqing Deng: data curation, formal analysis, writing – original draft, and visualization. Yuxuan Zhang: software. Lianjie Xu: data curation and methodology. Jinyu Lyu: data curation. Linyan Li: writing – review & editing. Fei Sun: conceptualization. Wen-Bin Zhang: writing – review & editing and methodology. Hanyu Gao: writing – review & editing and supervision.

## Conflicts of interest

There are no conflicts to declare.

## Supplementary Material

SC-016-D4SC08649J-s001
