# Supplementary material for: Computational discovery and systematic analysis of protein entangling motifs in nature: from algorithm to database
Source: Chem Sci. 2025 Mar 31;16(20):8998–9009. doi: 10.1039/d4sc08649j (PMC12013726; doi:10.1039/d4sc08649j)
Supplement: SC-016-D4SC08649J-s001 [file SC-016-D4SC08649J-s001.pdf]

Supplementary Information (SI) for Chemical Science.
This journal is © The Royal Society of Chemistry 2025

Supplementary Information

Computational Discovery and Systematic Analysis of Protein Entangling Motifs in Nature: From Algorithm to Database

Puqing Deng[1], Yuxuan Zhang[1], Lianjie Xu[2], Jinyu Lyu[1], Linyan Li[3], Fei Sun[1], Wen-Bin Zhang[2,4*], Hanyu Gao[1*]

[1]Department of Chemical and Biological Engineering, Hong Kong University of Science and Technology, Clear Water Bay, Hong Kong

[2]Beijing National Laboratory for Molecular Sciences, Key Laboratory of Polymer Chemistry & Physics of Ministry of Education, Center for Soft Matter Science and Engineering, College of Chemistry and Molecular Engineering, Peking University, Beijing 100871, P. R. China

[3]Department of Data Science, City University of Hong Kong, Kowloon, Hong Kong

[4]AI for Science (AI4S)-Preferred Program, Shenzhen Graduate School, Peking University, Shenzhen 518055, P. R. China

* To whom correspondence should be addressed. Email: hanyugao@ust.hk, wenbin@pku.edu.cn

Table S1. The number of entangling motifs left after each curation step.

	|GLN| & pLDDT	BSA	Core |GLN|	Symmetry	No topological links	Manual curation
**C2**	2145	2052	1617	1018	962	-
**C3**	2456	2269	1849	143	141	-
**Heterodimers**	299	226	137	-	130	12

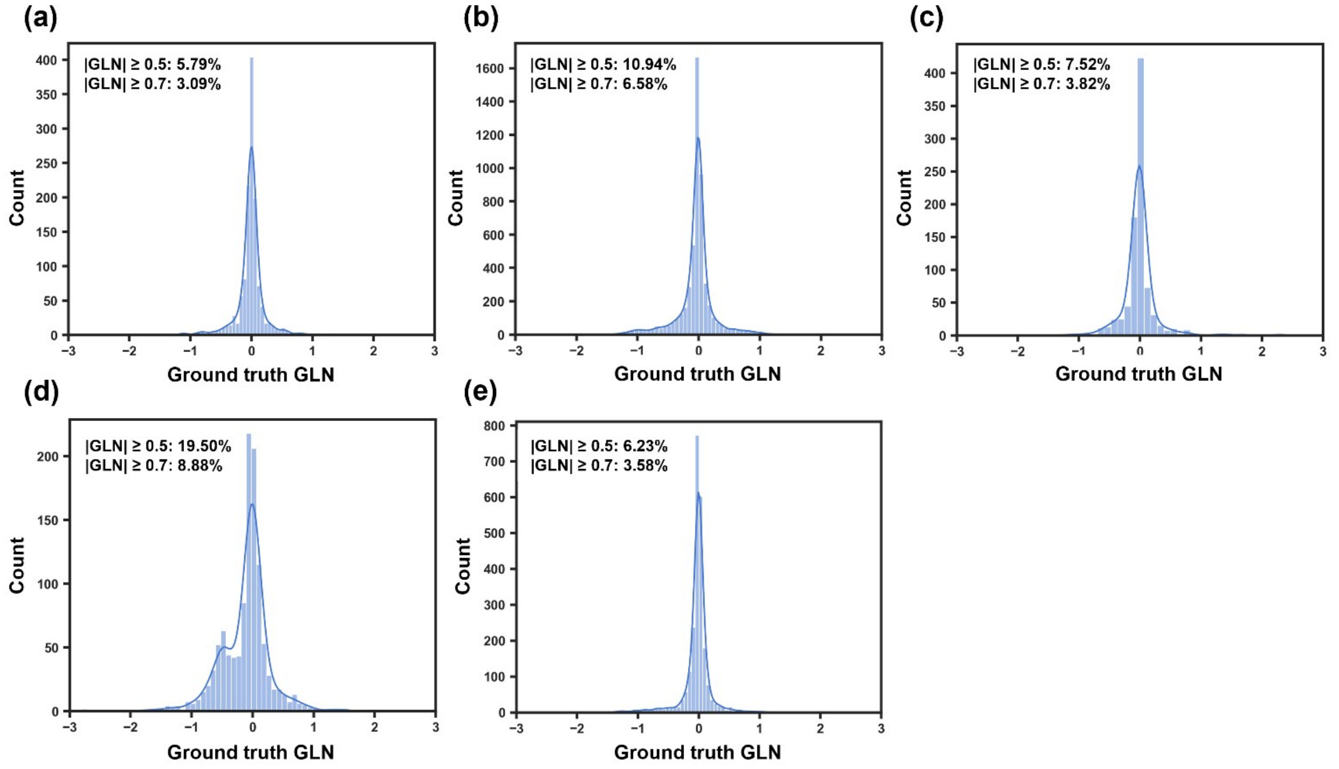

Figure S1. GLN distribution of different datasets. (a) PDB-Heterodim with 1295 assemblies; (b) PDB-C2 with 5302 assemblies; (c) PDB-C3 with 891 assemblies; (d) PDB-C4/D2 with 1149 assemblies; (e) PDB-Recent with 2375 assemblies.

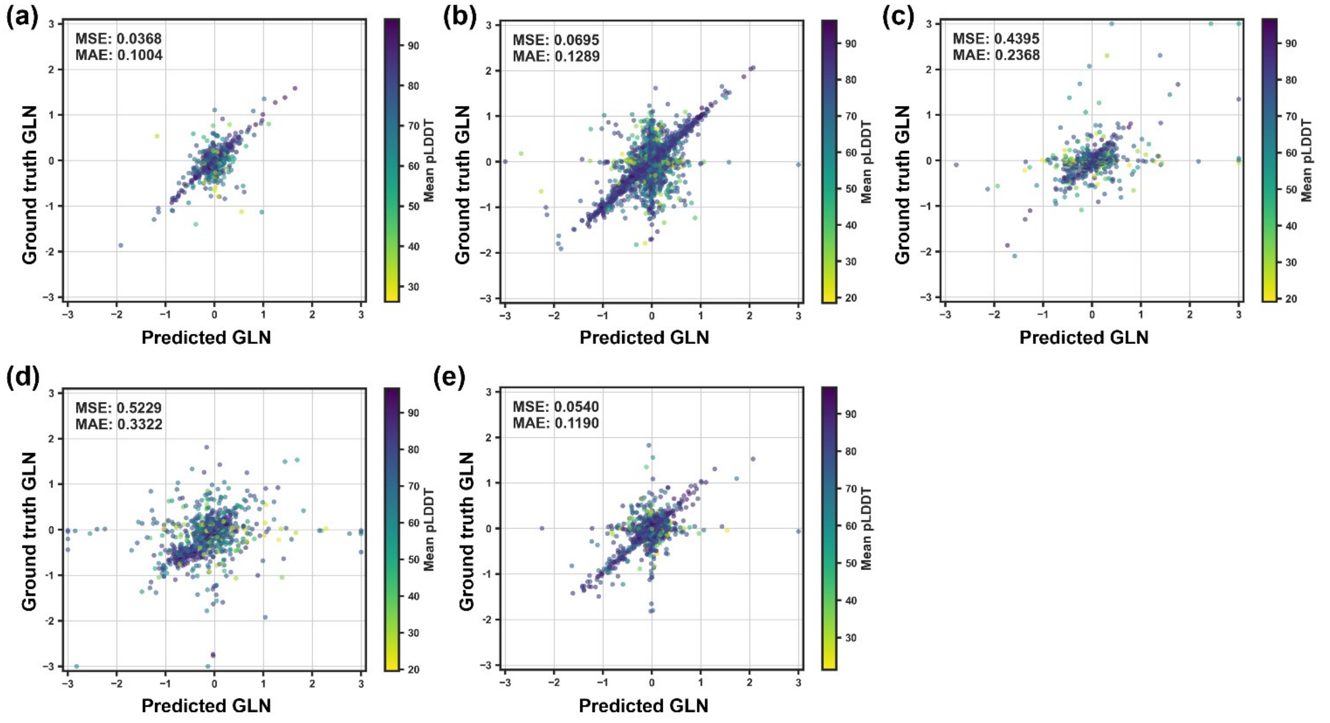

Figure S2. Detailed results of evaluating the performance of ESMFold in detecting entanglements in protein assembles for different datasets. (a) PDB-Heterodim; (b) PDB-C2; (c) PDB-C3; (d) PDB-C4/D2; (e) PDB-Recent

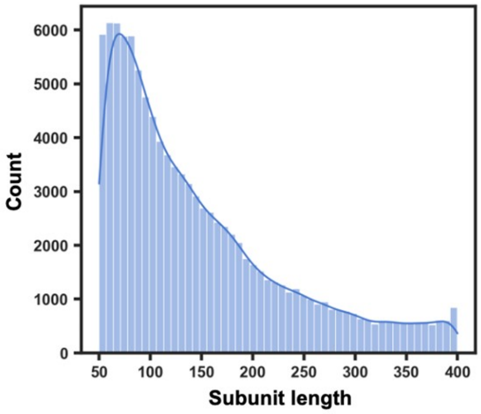

Figure S3. Length distribution of the sequences ready for screening.

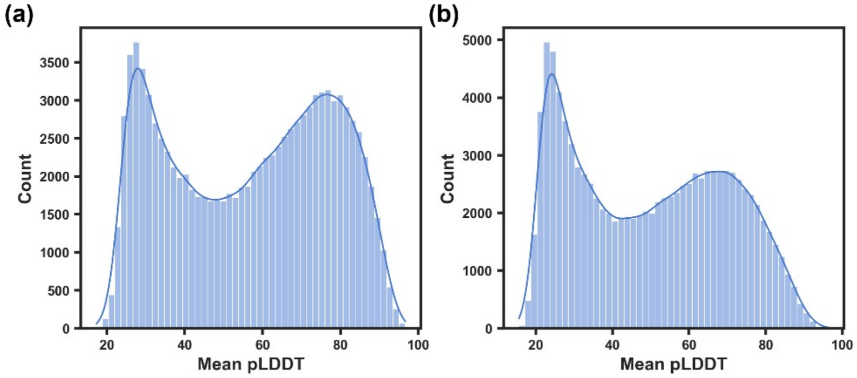

Figure S4. pLDDT distributions for sequence screening. (a) C2 entangling motif screening. (b) C3 entangling motif screening.

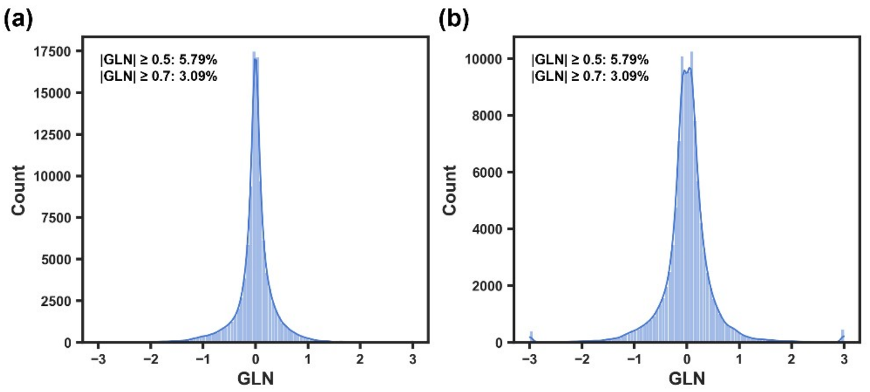

Figure S5. Predicted GLN distributions for sequence screening. (a) C2 entangling motif screening. (b) C3 entangling motif screening.

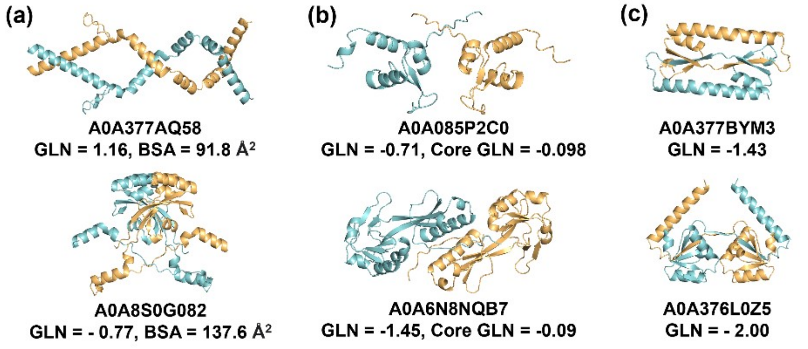

Figure S6. Examples of unqualified motifs we discarded in the filtration process, with (a) low BSA; (b) core |GLN| < 0.7; (c) unrealistic topological links.

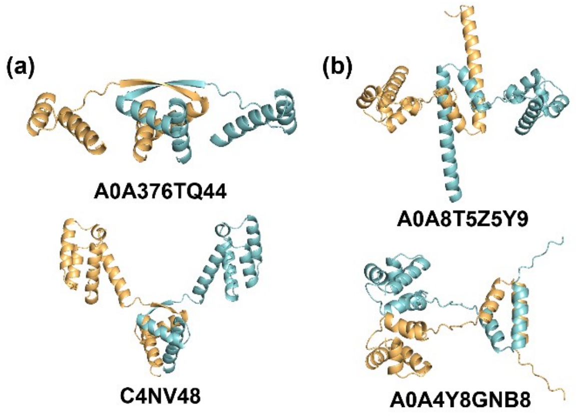

Figure S7. Motifs with similar entangled sites. (a) Ribbon-helix-helix entangled sites. (b) Entangled sites with two-helix bundles packed together.

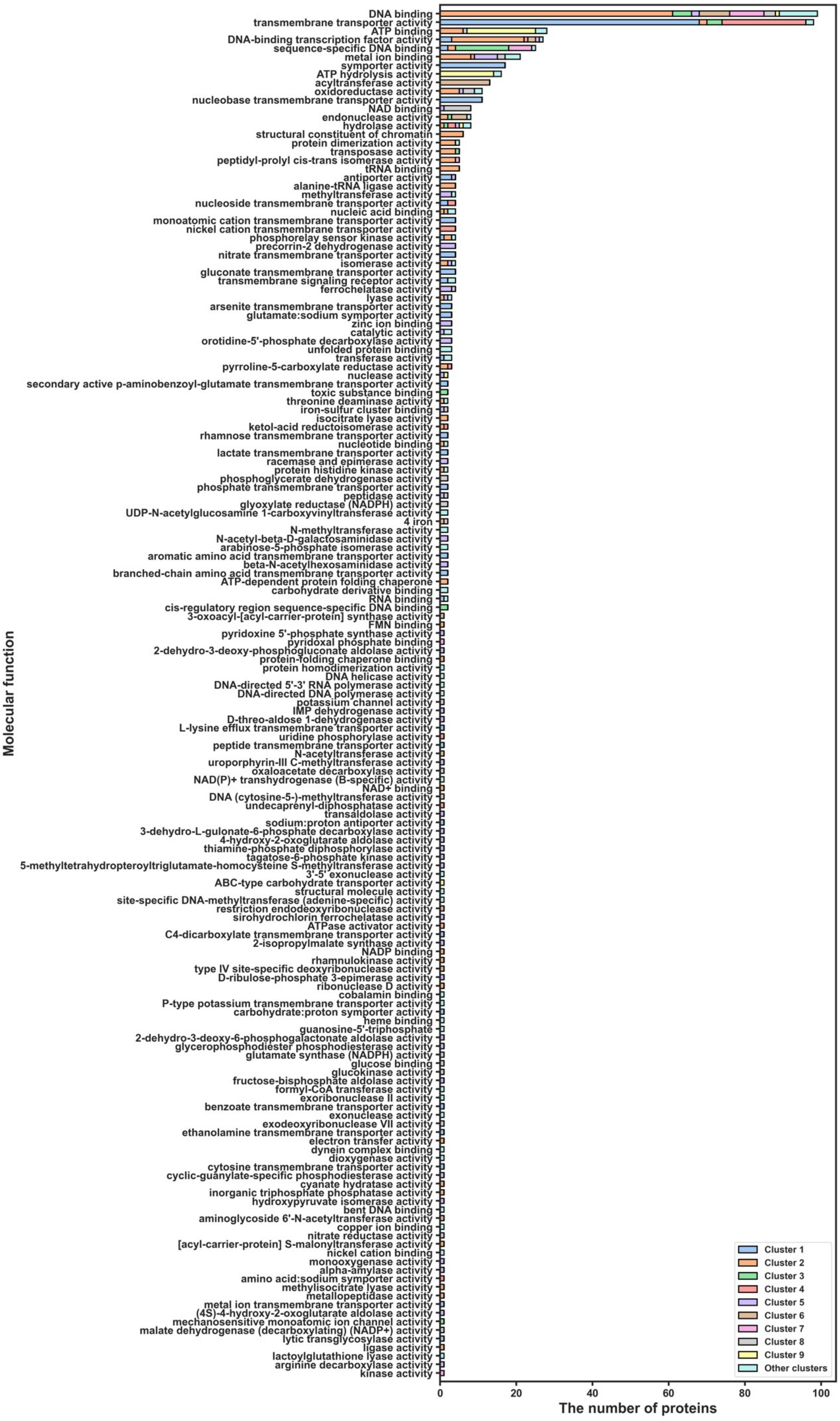
DNA binding
transmembrane transporter activity
ATP binding
DNA-binding transcription factor activity
sequence-specific DNA binding
metal ion binding
symporter activity
ATP hydrolysis activity
acyltransferase activity
oxidoreductase activity
nucleobase transmembrane transporter activity
NAD binding
endonuclease activity
hydrolase activity
structural constituent of chromatin
protein dimerization activity
transposase activity
peptidyl-prolyl cis-trans isomerase activity
tRNA binding
antiporter activity
alanine-tRNA ligase activity
methyltransferase activity
nucleoside transmembrane transporter activity
nucleic acid binding
monoatomic cation transmembrane transporter activity
nickel cation transmembrane transporter activity
phosphorelay sensor kinase activity
precorrin-2 dehydrogenase activity
nitrate transmembrane transporter activity
isomerase activity
gluconate transmembrane transporter activity
transmembrane signaling receptor activity
ferrochelatase activity
lyase activity
arsenite transmembrane transporter activity
glutamate:sodium symporter activity
zinc ion binding
catalytic activity
orotidine-5'-phosphate decarboxylase activity
unfolded protein binding
transferase activity
pyrroline-5-carboxylate reductase activity
nuclease activity
secondary active p-aminobenzoyl-glutamate transmembrane transporter activity
toxic substance binding
threonine deaminase activity
iron-sulfur cluster binding
isocitrate lyase activity
ketol-acid reductoisomerase activity
rhamnose transmembrane transporter activity
nucleotide binding
lactate transmembrane transporter activity
racemase and epimerase activity
protein histidine kinase activity
phosphoglycerate dehydrogenase activity
phosphate transmembrane transporter activity
peptidase activity
glyoxylate reductase (NADPH) activity
UDP-N-acetylglucosamine 1-carboxyvinyltransferase activity
4 iron
N-methyltransferase activity
N-acetyl-beta-D-galactosaminidase activity
arabinose-5-phosphate isomerase activity
aromatic amino acid transmembrane transporter activity
beta-N-acetylhexosaminidase activity
branched-chain amino acid transmembrane transporter activity
ATP-dependent protein folding chaperone
carbohydrate derivative binding
RNA binding
cis-regulatory region sequence-specific DNA binding
3-oxoacyl-[acyl-carrier-protein] synthase activity
FMN binding
pyridoxine 5'-phosphate synthase activity
pyridoxal phosphate binding
2-dehydro-3-deoxy-phosphogluconate aldolase activity
protein-folding chaperone binding
protein homodimerization activity
DNA helicase activity
DNA-directed 5'-3' RNA polymerase activity
DNA-directed DNA polymerase activity
potassium channel activity
IMP dehydrogenase activity
D-threo-aldose 1-dehydrogenase activity
L-lysine efflux transmembrane transporter activity
uridine phosphorylase activity
peptide transmembrane transporter activity
N-acetyltransferase activity
uroporphyrin-III C-methyltransferase activity
oxaloacetate decarboxylase activity
NAD(P)+ transhydrogenase (B-specific) activity
NAD+ binding
DNA (cytosine-5-)-methyltransferase activity
undecaprenyl-diphosphatase activity
transaldolase activity
sodium:proton antiporter activity
3-dehydro-L-gulonate-6-phosphate decarboxylase activity
4-hydroxy-2-oxoglutarate aldolase activity
thiamine-phosphate diphosphorylase activity
tagatose-6-phosphate kinase activity
5-methyltetrahydropteroyltriglutamate-homocysteine S-methyltransferase activity
3'-5' exonuclease activity
ABC-type carbohydrate transporter activity
structural molecule activity
site-specific DNA-methyltransferase (adenine-specific) activity
restriction endodeoxyribonuclease activity
sirohydrochlorin ferrochelatase activity
ATPase activator activity
C4-dicarboxylate transmembrane transporter activity
2-isopropylmalate synthase activity
NADP binding
rhamnulokinase activity
type IV site-specific deoxyribonuclease activity
D-ribulose-phosphate 3-epimerase activity
ribonuclease D activity
cobalamin binding
P-type potassium transmembrane transporter activity
carbohydrate:proton symporter activity
heme binding
guanosine-5'-triphosphate
2-dehydro-3-deoxy-6-phosphogalactonate aldolase activity
glycerophosphodiester phosphodiesterase activity
glutamate synthase (NADPH) activity
glucose binding
glucokinase activity
fructose-bisphosphate aldolase activity
formyl-CoA transferase activity
exoribonuclease II activity
benzoate transmembrane transporter activity
exonuclease activity
exodeoxyribonuclease VII activity
ethanolamine transmembrane transporter activity
electron transfer activity
dynein complex binding
dioxygenase activity
cytosine transmembrane transporter activity
cyclic-guanylate-specific phosphodiesterase activity
cyanate hydratase activity
inorganic triphosphate phosphatase activity
hydroxypyruvate isomerase activity
bent DNA binding
aminoglycoside 6'-N-acetyltransferase activity
copper ion binding
nitrate reductase activity
[acyl-carrier-protein] S-malonyltransferase activity
nickel cation binding
monooxygenase activity
alpha-amylase activity
amino acid:sodium symporter activity
methylisocitrate lyase activity
metallopeptidase activity
metal ion transmembrane transporter activity
(4S)-4-hydroxy-2-oxoglutarate aldolase activity
mechanosensitive monoatomic ion channel activity
malate dehydrogenase (decarboxylating) (NADP+) activity
lytic transglycosylase activity
ligase activity
lactoylglutathione lyase activity
arginine decarboxylase activity
kinase activity
Molecular function
0
20
40
60
80
100
The number of proteins
Cluster 1
Cluster 2
Cluster 3
Cluster 4
Cluster 5
Cluster 6
Cluster 7
Cluster 8
Cluster 9
Other clusters

Figure S8. Complete GO annotation distribution of C2 entangling motifs.

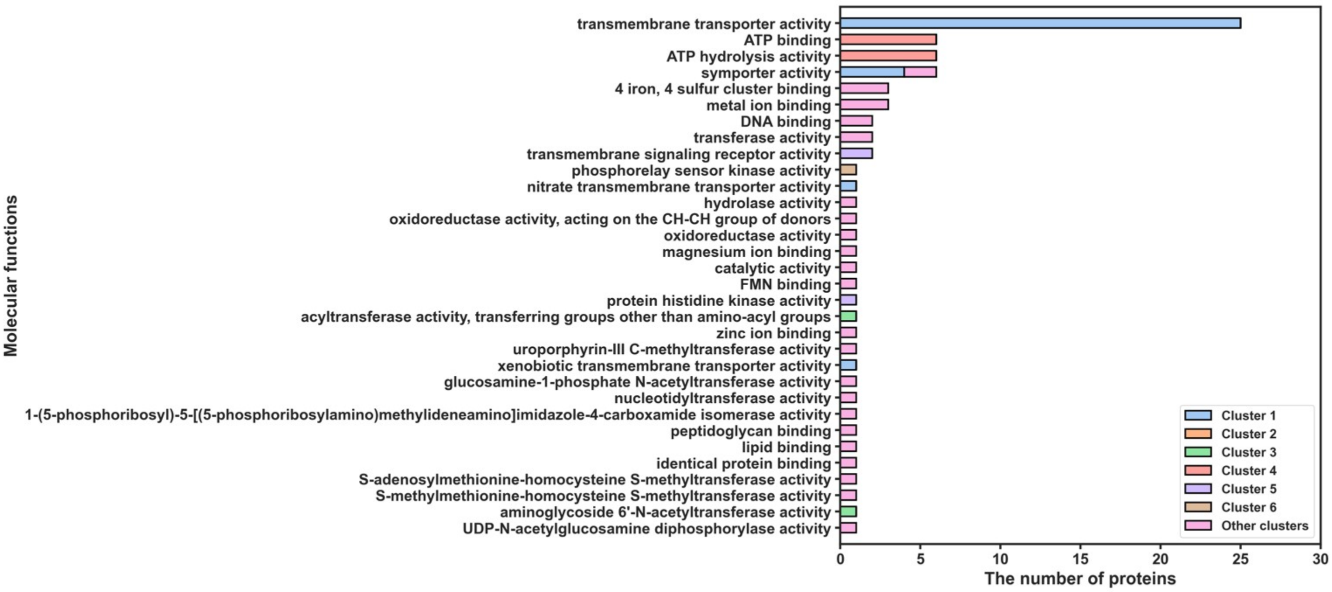

Figure S9. Complete GO annotation distribution of C3 entangling motifs.

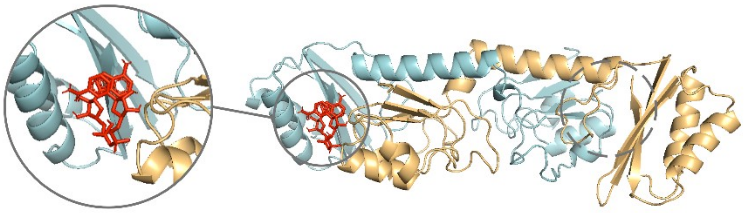

Figure S10. Molecular docking result of NAD and a NAD binding entangling motif (accession number: A0A2X1PF19) by AutoDock. Inferred by symmetry, another NAD molecule can be also docked into the right pocket.
